# Update on the Phylodynamic and Genetic Variability of Marburg Virus

**DOI:** 10.3390/v15081721

**Published:** 2023-08-11

**Authors:** Fabio Scarpa, Liliana Bazzani, Marta Giovanetti, Alessandra Ciccozzi, Francesca Benedetti, Davide Zella, Daria Sanna, Marco Casu, Alessandra Borsetti, Eleonora Cella, Stefano Pascarella, Antonello Maruotti, Massimo Ciccozzi

**Affiliations:** 1 Department of Biomedical Sciences, University of Sassari, 07100 Sassari, Italy; darsanna@uniss.it; 2 Department of Science and Technology for Humans and the Environment, Università Campus Bio-Medico di Roma, 00128 Rome, Italy; liliana.bazzani@alcampus.it (L.B.); giovanetti.marta@gmail.com (M.G.); 3 Instituto Rene Rachou, Fundação Oswaldo Cruz, Belo Horizonte 30190-009, MG, Brazil; 4 Unit of Medical Statistics and Molecular Epidemiology, Università Campus Bio-Medico di Roma, 00128 Rome, Italy; ale_ciccozzi97@icloud.com (A.C.); m.ciccozzi@unicampus.it (M.C.); 5 Institute of Human Virlogy and Global Virusn Network Center, Deparment of Biochemistry and Molecular Biology, University for Maryland School of Medicine, Baltimore, MD 21201, USA; fbenedetti@som.umaryland.edu (F.B.); dzella@som.umaryland.edu (D.Z.); 6 Department of Veterinary Medicine, University of Sassari, 07100 Sassari, Italy; marcasu@uniss.it; 7 National HIV/AIDS Research Center (CNAIDS), National Institute of Health, 00161 Rome, Italy; alessandra.borsetti@iss.it; 8 Burnett School of Biomedical Sciences, University of Central Florida, Orlando, FL 32816, USA; eleonora.cella@yahoo.it; 9 Department of Biochemical Sciences “A. Rossi Fanelli”, Sapienza Università di Roma, 00185 Rome, Italy; stefano.pascarella@uniroma1.it; 10 Department GEPLI, Libera Università Maria Ss Assunta, 00193 Rome, Italy

**Keywords:** MARV, genetic variability, genomic surveillance, phylodynamics, Africa

## Abstract

The COVID-19 pandemic has not only strained healthcare systems in Africa but has also intensified the impact of emerging and re-emerging diseases. Specifically in Equatorial Guinea, mirroring the situation in other African countries, unique zoonotic outbreaks have occurred during this challenging period. One notable resurgence is Marburg virus disease (MVD), which has further burdened the already fragile healthcare system. The re-emergence of the Marburg virus amid the COVID-19 pandemic is believed to stem from a probable zoonotic spill-over, although the precise transmission routes remain uncertain. Given the gravity of the situation, addressing the existing challenges is paramount. Though the genome sequences from the current outbreak were not available for this study, we analyzed all the available whole genome sequences of this re-emerging pathogen to advocate for a shift towards active surveillance. This is essential to ensure the successful containment of any potential Marburg virus outbreak in Equatorial Guinea and the wider African context. This study, which presents an update on the phylodynamics and the genetic variability of MARV, further confirmed the existence of at least two distinct patterns of viral spread. One pattern demonstrates a slower but continuous and recurring virus circulation, while the other exhibits a faster yet limited and episodic spread. These results highlight the critical need to strengthen genomic surveillance in the region to effectively curb the pathogen’s dissemination. Moreover, the study emphasizes the importance of prompt alert management, comprehensive case investigation and analysis, contact tracing, and active case searching. These steps are vital to support the healthcare system’s response to this emerging health crisis. By implementing these strategies, we can better arm ourselves against the challenges posed by the resurgence of the Marburg virus and other infectious diseases.

## 1. Introduction

Throughout the COVID-19 pandemic, numerous African countries, including Equatorial Guinea, experienced distinct zoonotic outbreaks [1]. Importantly, this global health crisis has further strained the already fragile healthcare system, exacerbating the impact of both emerging and re-emerging diseases, such as the recent Marburg virus (MARV) disease outbreak in this region. In particular, the re-emergence of MARV during the COVID-19 pandemic is likely attributed to zoonotic spill-over events [2]. The Marburg virus belongs to the Filoviridae family (*Filovirus*) and holds the distinction of being the first *Filovirus* ever discovered [2]. It is an enveloped, single-stranded, negative-sense RNA virus, ranging in length from 800 nm to 14,000 nm. MARV, together with the Ravn virus (RAVV) [3], is the causative agent of Marburg virus disease (MVD), a highly lethal illness [4]. While the virus is primarily transmitted from animals to humans, it can also spread through direct contact with infected individuals’ blood, secretions, organs, and bodily fluids, as well as contaminated surfaces and materials like bedding and clothing [5]. The Egyptian fruit bat, *Rousettus aegyptiacus* of the Pteropodidae family, serves as the natural host of MARV and RAVV. Human infection with MVD is often associated with prolonged contact with mines or caves inhabited by bat colonies [5]. The World Health Organization (WHO) has recognized MARV as an exceptionally significant global pathogen, categorizing it as a risk group 4 pathogen with a high case fatality rate (CFR) ranging from 24% to 88% [6]. Since its discovery in 1967, there have been 14 reported MVD outbreaks, predominantly in sub-Saharan Africa, including the Democratic Republic of Congo, Kenya, Angola, Uganda, and South Africa [4]. On 13 February 2023, the Ministry of Health and Social Welfare (MOW) of Equatorial Guinea officially declared the first-ever outbreak of MVD in two villages, Ngum-Esatop and Evusoc Mokomo, located in the Nsok-Nsomo district of the Kie-Nterm province [6]. As of 1 May 2023, a total of 17 laboratory-confirmed cases of MVD and 23 probable cases have been reported. Among the laboratory-confirmed cases, there have been 12 deaths, resulting in a CFR of 75% [6]. Confirmed or probable cases have been reported in five districts: Bata, Ebeibiyin, Evinayong, Nsok Nsomo, and Nsork, spanning four of the country’s eight provinces (Centro Sur, Kié-Ntem, Litoral, and Wele-Nzas) [7]. Bata, located in the Litoral province, has been the most affected district, with 11 laboratory-confirmed MVD cases reported. While some reported cases are connected within a social network or through proximity, the presence of cases and clusters across multiple districts without clear epidemiological links suggests potential undetected virus transmission [8]. The incubation period of MVD varies from 2 to 21 days, and the clinical presentation of the illness typically includes abrupt onset of high fever, severe headache, and profound malaise. Severe hemorrhagic manifestations may occur between five and seven days after the onset of symptoms [8]. Diagnosing and treating MVD pose significant challenges, as the early stages of the disease closely resemble other infectious diseases such as malaria, shigellosis, meningitis, typhoid fever, and other viral hemorrhagic fevers [6]. Moreover, there are currently no approved vaccines or therapeutics for MARV. Additionally, the sporadic nature of outbreaks poses challenges in testing new countermeasures during these crises, both in terms of ethical considerations and logistical complexities [9]. In this study, given the lack of genome sequences from the ongoing outbreak, we analyzed all available whole genome sequences of this re-emerging virus from public databases such as the NCBI Virus portal [10]. Indeed, understanding the genetic variability of a virus is of paramount importance for constant monitoring and effective management of new waves of infections. Viruses typically have the ability to mutate over time and these mutations can affect the virus’s transmission rate, disease severity, and the efficacy of available treatments and vaccines.

Moreover, a continuous and uninterrupted surveillance of viral genetic variations enables scientists and public health officials to trace the emergence and spread of new viral strains. Additionally, monitoring genetic diversity facilitates the identification of clusters or subtypes of the virus circulating in different regions. This knowledge helps in understanding transmission patterns and guiding targeted public health interventions. Our results strongly advocate for active surveillance to ensure the effective containment of any potential outbreak of this re-emerging viral pathogen, not only in Equatorial Guinea but also throughout the African continent. Enhancing genomic surveillance in the country, coupled with measures like alert management, case investigation, contact tracing, and active case search, plays a pivotal role in bolstering the health care system’s response to this emerging health crisis. 

## 2. Materials and Methods

### Phylodynamics

To investigate the evolutionary relationship of Marburg virus over time, a dataset containing all genomes belonging to MARV has been built, downloading all genomes currently available in NCBI Virus portal (https://www.ncbi.nlm.nih.gov/labs/virus/vssi/#/, accessed on 15 June 2023) [10]. The dataset consisted of a total of 86 complete genomes, comprising strains from 11 countries. 

Phylogenetic reconstruction was performed by following Scarpa et al. [11]. The genomes were aligned using the L-INS-I algorithm implemented in Mafft 7.471 [12] and manually checked and edited with Unipro UGENE v.35 [13]. The best probabilistic model of genome evolution was determined using jModeltest 2.1.1 [14] through a maximum likelihood optimized search. Evolutionary relationships among lineages were examined using MrBayes 3.2.7 [15]. Two independent runs were conducted, each consisting of four Metropolis-coupled Markov-chain Monte Carlo (MCMCMC) simulations, with one cold and three heated chains. These runs were performed simultaneously for 5,000,000 generations, with trees sampled every 1000 generations. The first 25% of the 10,000 sampled trees were discarded as burn-in. Nodes with a posterior probability greater than 0.95 were considered statistically supported. The resulting phylogenetic tree was visualized using FigTree 1.4.0 [16].

To obtain the time-calibrated tree, to determine the evolutionary relationship among variants and estimate the time of divergence, we employed Bayesian Inference (BI) with the software BEAST 1.10.4 [17]. This kind of analysis allows, by applying the neutral theory of molecular evolution, the performance of minimum age molecular dating, where the branch lengths are proportional to the elapsed time. The analysis involved runs of 200 million generations using various demographic and clock models. To determine the best model for dating inferences, we compared strict and uncorrelated log-normal relaxed clock models. These clock models were further assessed under different parametric demographic models (constant population size, exponential population growth, and expansion population growth) and a piecewise-constant model (Bayesian skyline). Model selection was conducted using the Bayes Factor test [18], comparing the 2lnBF of Marginal Likelihoods values, using the software Tracer 1.7 [19], as described in Mugosa et al. [20]. Only values of effective sample size (ESS) ≥ 200 were considered during the screening process. The maximum clade credibility tree was generated and annotated using the TreeAnnotator software from the BEAST package. The resulting phylogenetic trees were edited and visualized using FigTree 1.4.0 [16]. To reconstruct the Bayesian skyline plot (BSP), a further analyses involving runs of 200 million generations under the Bayesian skyline model with the uncorrelated log-normal relaxed clock model was performed. 

To identify possible subgroups within genetic clusters and assess the genetic variability among genomes, a principal coordinate analysis (PCoA) was conducted using GenAlEx 6.5 [21]. The PCoA reconstruction was based on a pairwise p-distance matrix of genetic data. The aim of this analysis was to examine the dissimilarity represented by the genetic variability among the analyzed genomes.

## 3. Results

**Figure 1:**
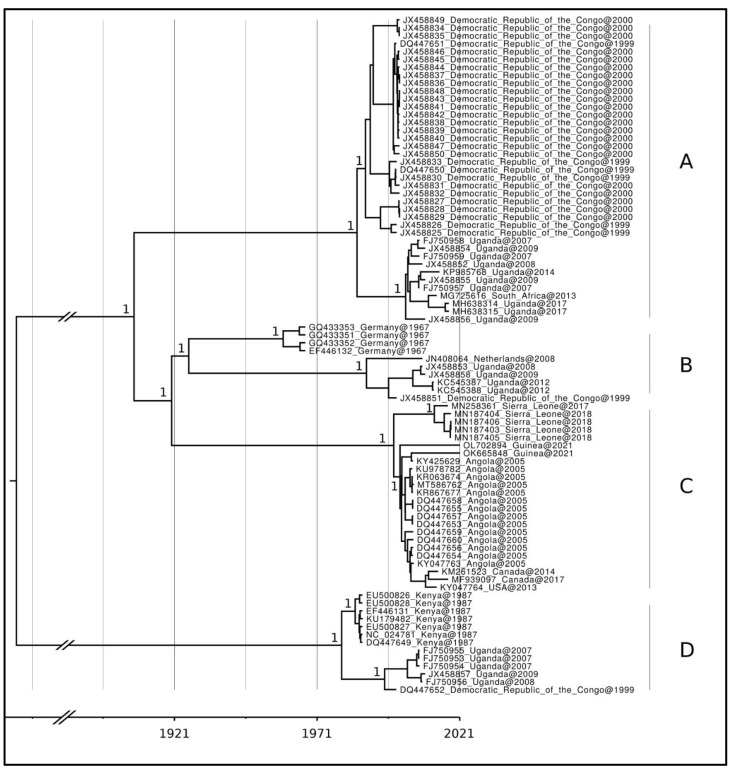
Phylogenetic reconstruction of MARV. Time-scaled maximum clade credibility phylogenetic tree of *n* = 86 MARV genome sequences available on NCBIVirus as of 11 June 2023. Values around key nodes represent posterior probability support. A–D represent the main clades found in the phylogenetic reconstruction.

The Bayes Factor test indicated that the coalescent constant size, under the lognormal uncorrelated relaxed clock model, provided a significantly better fit to the data compared to other tested models (2lnBF = 11.2). The phylogenetic tree obtained with MrBayes and the time-scaled phylogenetic tree obtained by using the software Beast provide the same topology; therefore, only the latter is showed (Figure 1). The Bayesian evolutionary tree, obtained from a dataset containing all available genomes of Marburg virus (Figure 1), reveals well-supported branches (posterior probabilities = 1). Overall, the tree exhibits a distinct and statistically significant genetic structure, geographically and time based. Following the application of midpoint rooting, the tree reveals two well-separated clades, one representative of Marburg virus (MARV) and one of Ravn virus (RAVV). The first clade (MARV) comprises a diverse assemblage of discrete groups represented by three clusters, while the second (RAVV) was represented by only one cluster. More specifically, regarding the first main clade (clusters A + B + C in the tree), cluster A is composed of genomes from: (i) the Demographic Republic of the Congo 1999–2000), (ii) South Africa (only one sampled in 2013) and (iii) Uganda (2007–2014). In turn, this cluster presents a sister clade relationship with the heterogeneous sub-clade comprising the clusters B + C, composed of genomes from Germany, Netherlands, Uganda, Demographic Republic of the Congo, Sierra Leona, Angola, Guinea, USA and Canada. More specifically, cluster B is composed of genomes form Germany (1967), Netherlands (2008), Uganda (2008–2012) and Demographic Republic of the Congo (1999). This cluster in turn shows a sister clade relationship with cluster C that is composed of genomes from Sierra Leone (2017–2018), Guinea (2021), Angola (2005), Canada (2014–2017) and USA (2013). The second main clade is represented by one cluster composed of genomes from Kenya (1987), Uganda (2007–2009) and Demographic Republic of the Congo (1999). The root of the tree is placed about 230 years ago and the branches supporting the two main clades are indicate a length of 116 and 190 years for MARV and RAVV, respectively. The Time of the Most Recent Common Ancestor (TMRCA) of the first main clade dates back to about 114 years ago while the second main clade is about 42 years old. Within the clades, the clusters are dated as follows, A: 36 years old (CI95%HPD = 27–52 years), B: 95 years old (CI95%HPD = 64–112 years), C: 23 years old (CI95%HPD = 22–38 years), D: 41 years old (CI95%HPD = 35–75 years).

**Figure 2:**
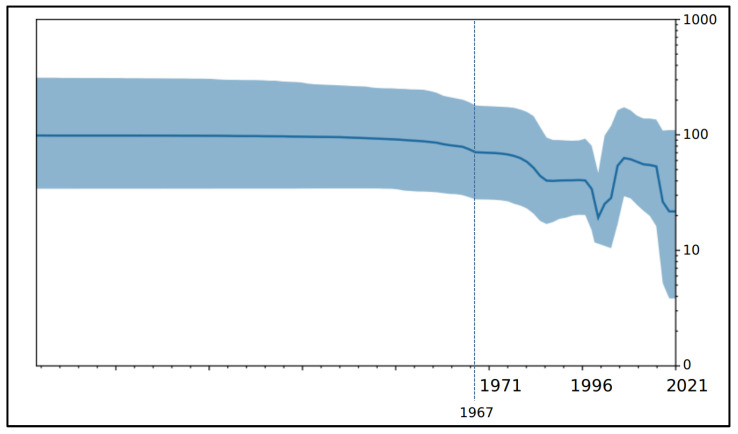
Bayesian skyline plot of MARV. The viral effective size (y-axis) is shown as a function of time (x-axis). The solid area represents the 95% high posterior density (HPD) region.

The Bayesian skyline plot (BSP) graph (Figure 2) depicts the genetic variability (y-axis) as a function of time (x-axis). Since genetic variability is directly linked to the viral population size, it enables us to observe variations in the viral population size over time. The BSP shows an initial flattened genetic variability with a decrease in viral population size during the 2000s. Following this period, genetic variability, and consequently the viral population size, surged and reached a plateau between 2005 and 2015. Since that point, a subsequent decline has been observed, persisting into the current period. Oscillations in genetic variability represented in the graph reflect the diversification followed in the tree by recent clade. 

**Figure 3:**
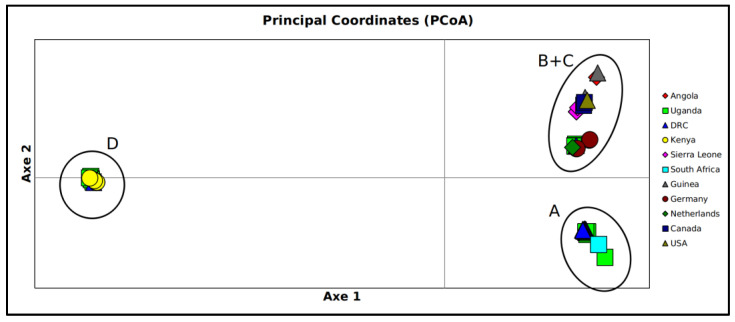
Principal coordinates analysis performed on all MARV genomes datasets. Bidimensional plot shows the genetic differentiation among genomes due to the nucleotide substitutions per site found in the dataset. Capital letters in the plot indicate the genetic clusters labeled in the tree. The total variability explained by the first three axes amounts to 97.67% (Axe 1: 87.75; Axe 2: 7.46; Axe 3: 2.75).

Analyses of principal coordinates (PCoA) (Figure 3), based on genetic data, depicts a representation of the genetic relatedness and variability among samples. In the graph, each genome is depicted as a data point, and the relative positions of these points reflect the genetic similarity or difference between samples. Samples that appear close together indicate genetically related groups; conversely, samples that are widely scattered across the graph signify higher genetic diversity and heterogeneity among the samples. 

PCoA (Figure 3) revealed the same primary subdivision as observed through midpoint rooting, accounting for 87.75% of variability explaining this division (Axis 1). In the PCoA, evolutionarily close clades that appeared in the tree as sister groups clustered together, forming three main groups. The total variability explained by the first three axes amounts to 97.67% (Axis 1: 87.75; Axis 2: 7.46; Axis 3: 2.75). 

## 4. Discussion

Human-to-human transmission of Marburg virus (MARV) occurs from direct contact (through skin lesions or mucous membranes) with blood, secretions, organs, or other body fluids of infected persons and with surfaces and materials (e.g., bedding, clothing) contaminated with these fluids; health care workers can also be infected while treating patients with suspected or confirmed MVD. Indeed, the first case of the MVD was reported in 1967 when lab personnel working with African green monkeys became infected in Germany and Serbia simultaneously. Burial ceremonies involving direct contact with the body of the deceased may also contribute to transmission of Marburg virus. The incubation period ranges from 2 to 21 days, and MVD starts abruptly, with high fever, headache, and severe malaise. Severe hemorrhagic manifestations appear between five and seven days after the onset of symptoms, and fatal cases usually have some form of bleeding, often from multiple areas. Supportive care (rehydration with oral or intravenous fluids) and the treatment of specific symptoms improve survival [22]. Despite interest in and progress toward deploying monoclonal antibodies [23], direct antivirals, and small interfering RNA (siRNA) molecules [24,25], no currently approved treatment for MVD is currently available. Indeed, in many ways, the therapeutic approach in 2020 is not dissimilar to the one taken in Belgrade in 1967 following the first outbreak. Variations in case fatality rates between resource-constrained settings and those with greater capacity indicate the potential impact of healthcare provisions on disease outcomes [26,27]. However, much like with Ebola virus diseases, substantial evidence to inform comprehensive supportive treatment guidelines remains insufficient, even as of 2020. In February 2023, Equatorial Guinea’s Ministry of Health and Social Welfare declared an outbreak of MVD after deaths from suspected viral hemorrhagic fever were reported between January and February, 2023, Since the outbreak declaration and through June 2023, 17 confirmed and 23 probable cases have been reported in the mainland region of Equatorial Guinea. Twelve of the confirmed cases have died and all probable cases have died (the mortality rate among confirmed cases is 75%, excluding one confirmed case for which the outcome is unknown). On June 2023, after two consecutive incubation periods (42 days) without reporting any new confirmed case, the Ministry of Health of Equatorial Guinea declared the end of the epidemic. This is the first time Equatorial Guinea has reported an outbreak of MVD. Another MVD outbreak was recently declared in the United Republic of Tanzania (WHO Communiqué of June, 2023). Other MVD outbreaks have been previously reported in Ghana (2022), Guinea (2021), Uganda (2017, 2014, 2012, 2007), Angola (2004–2005), Democratic Republic of Congo (1998 and 2000), Kenya (1990, 1987, 1980), and South Africa (1975). Here, we conducted an updated analysis of the phylodynamics and genetic variability of MARV using all available genomes spanning different time periods. Unfortunately, the most recent isolate is from 2021 and genomes belonging to the outbreak of 2023 are not still available. We attempted to explore and identify any potential differences over time between these epidemics, utilizing evolutionary and phylogenetic tools. All sequences available in the gene bank were employed. Our findings highlighted that the population dynamics of MARV illustrate a general evolutionary trajectory characterized by modest levels of genetic variability and slow evolutionary rate. This perspective is further supported by the phylogenomic reconstruction, which reveals the existence of several small clusters largely independent of one other. In certain instances, it seems that specific specimens from a particular cluster branch out to interact with other clusters, forming a more expansive epidemic-type cluster within a closed community. This pattern closely resembles the seasonal flu, as observed in studies such as Mugosa et al. [20]. Recently, dynamics of similar nature have been observed in the monkeypox virus [28]. In general, the phylogenetic tree displays a distinct and statistically significant genetic structure based on both geographical and temporal factors. The presence of two primary, distinctly separated clades, confirms that MARV and RAVV are two evolutionary related yet quite divergent strains (with a nucleotide divergence lower than 20%). 

This pattern has also been substantiated by the PCoA graph, which fully supports the geographical structuring, identifying three distinct groups that align with the main clades observed in the tree (clade A, clades B + C, and clade A). Interestingly, genomes from both Uganda and the Democratic Republic of the Congo are present in each cluster, although the Democratic Republic of the Congo genomes are represented by only a single genome in two of the clusters. As for the genomes from Uganda, they are well represented in all of the main clusters, including ones from relatively recent sampling dates (i.e., 2017). The largest distance depicted in the graph is between the cluster D (RAVV) and the clusters A + B + C. Indeed, they share a common ancestor dated back about 230 years ago with broad range of credibility interval, as found in previous studies [29,30]. Such a condition is typical when there is missing information in the dataset or incomplete steps in the process of reconstructing the evolutionary history of the two lineages. Concerning the dating of MARV, its ancestor dates back 114 years, suggesting that MARV had been present in an enzonotic cycle involving non-human hosts for a substantial period before its initial documentation in humans. The branch supporting the diversification of cluster A spans 78 years. This condition may represent a lineage with a prolonged existence and probable frequent recurrence, as confirmed by short branches within internal clades. These findings, along with their high level of nucleotide similarity (also depicted by their distance in the PCoA) and their heterogeneous isolation dates, suggest that these lineages may have the potential to periodically emerge in reservoir animals and become dominant in different locations due to the continuous and uninterrupted viral evolution, primarily influenced by genetic drift. The common ancestor shared by clusters B and C dates back about 100 years. Subsequently, cluster B and C are dated back 95 and 23 years, respectively. Cluster B contains the strain that caused the first known outbreak in Europe in 1967, and considering that the common ancestor to genomes from Germany dates back about five years before (1962), this further confirms the hypothesis of the enzonotic cycle involving non-human hosts previously described. Cluster C, which has a relatively recent common ancestor dating back around 23 years, contains genomes from Angola, Sierra Leone, Guinea, and North America, and it appears to be the cluster with the faster evolution. Based off its internal sub-structuring, confirmed both by the tree and the PCoA, it could be speculated that this cluster consists of lineages that, after emerging, remain confined to a specific episode in terms of both time and location and seem to disappear shortly thereafter. This last one may be the more potentially dangerous clade. Overall, the heterogeneous and complex scenario portrayed by the reconstructed phylogeny may represent multiple spillovers from the animal reservoir, as previously described for MARV by Bausch et al. [31]. 

In general, the BSP displays a flattened genetic variability. The genetic distances matrix further supports this observation, as the largest value of genetic distance within the internal clusters was found to be 0.003 (±0.0001). Specifically, the BSP graph displayed an initial prolonged phase of flattened genetic variability, with no variations accompanied by a decrease in the viral population size during the 2000s. Afterwards, there was an increase in genetic variability and, consequently, the viral population size reached a plateau between 2005 and 2015. However, a subsequent decrease in genetic variability took place, continuing up to the present day. The fluctuations in genetic variability depicted in the graph reflect the diversification observed in the tree, particularly in relation to the more recent clades. Such fluctuations are the result of localized outbreaks occurring over time. Typically, during an outbreak, the genetic variability and viral population size rise until reaching a plateau and then decrease again. Ever since the first documented case in 1967, numerous outbreaks of MVD have occurred, following initial contact with wild animals and contraction of the disease. Despite trials with a wide range of medications to date, no concrete solution has been achieved. As noted, the most reliable method of treatment remains supportive care with careful monitoring and isolation of the patient. Disease-modifying drugs and inhibitors of viral protein have shown some promising results in many patients and can be administered to those affected; however, they might not be the final answer to this deadly virus. Vaccine research likely remains a more reliable option for creating a vaccine to protect populations especially vulnerable to the disease. With the discovery of the filoviruses now over four decades old, one could question why there is still no approved vaccine available for human use. While several experimental vaccine approaches have been tested for Ebola virus and, to a lesser extent, for MARV, no progress seems to have been made towards clinical trials to date. Given that filoviruses can only be handled in high-containment facilities available in a few countries world-wide (biosafety level 4, BSL-4) and that, until 2014, they had not caused more than a few thousand human fatalities, there was likely never sufficient commercial interest or funding available for the development of licensed countermeasures. The situation changed following the EBOV epidemic that devastated West Africa from 2013 to 2016; clinical trials for the most promising countermeasure approaches were accelerated and funding was made available for the licensure process. The problem now is not waiting until a devastating Marburg epidemic occurs in some African regions or worldwide. In light of the recent major and lethal outbreaks in Africa regions, it is crucial for health authorities to formulate a clear and applicable pandemic strategy for the current times, where an uncontrolled endemic and pandemic can rapidly escalate an epidemic, causing chaos for health facilities already strained by numerous challenges. 

In this context, it should be highlighted that understanding the genetic variability and evolutionary patterns of viruses has become increasingly vital in the context of constant monitoring and effective public health surveillance, for instance see Nelson et al. [32] for Influenza A Virus. Recent advancements in genomic technologies have provided researchers with invaluable insights into the dynamics of viral pathogens, allowing for the precise tracking of their evolution and transmission [33]. Genetic variability within a virus population offers critical information about its adaptability, virulence, and transmission potential. Analyzing the genetic makeup of a virus helps identify distinct strains and track their prevalence over time, enabling the anticipation of potential changes in virulence or drug resistance and the design of appropriate control measures. Moreover, studying the evolutionary patterns of a virus provides valuable insights into its transmission dynamics (see, e.g., Scarpa et al. [34] and references therein). By comparing genetic sequences from different cases, it is possible to reconstruct the transmission network and identify clusters of related infections. This knowledge is invaluable for understanding the routes of transmission and identifying potential sources of outbreaks [35]. By integrating genetic data into public health surveillance systems, authorities can establish a comprehensive and dynamic approach to monitoring infectious diseases. Moreover, the one health approach should be applied, which recognizes the interdependence of human, animal, and environmental health [36]. By considering the health of all these components together, public health efforts can become more effective in identifying potential sources of outbreaks and predicting disease emergence [36]. Traditional surveillance methods often rely on epidemiological data alone, which can be limited in their ability to detect emerging threats. Genetic data complement these methods, providing a more comprehensive picture of the pathogen’s behavior (see Ling-Hu et al. [37]). Accordingly, embracing genomic technologies as a standard surveillance tool allows for early detection, precise monitoring, and effective control of infectious diseases. By leveraging genetic data, public health authorities can proactively safeguard communities, respond swiftly to outbreaks, and optimize resource allocation to protect global health.

## 5. Conclusions

In conclusion, it is crucial to continuously monitor the genetic variability of viruses to inform proactive and evidence-based public health interventions. By being alert and flexible in response to viral mutations, we can successfully navigate and respond to novel waves of infections, minimizing their impact and preserving population health. In this context, it is essential to promptly gather current data related to the virus resurgence in Equatorial Guinea and to determine the current cluster. Once the samples of 2023 are accessible, it becomes crucial to sequence them, and analyze and incorporate them into existing studies. This enables us to determine the group of belonging and their potential for spread, thus enhancing our understanding of the virus’s behavior and transmission patterns. Analyzing all available genomes over times, we found at least two different specific patterns of viral spread. One pattern demonstrates a slower, yet persistent and recurring, virus circulation, while the other showcases a faster but episode-confined spread. Understanding these patterns helps to provide a comprehensive view of the present status of Marburg virus (MARV) diffusion. Regular genomic surveillance coupled with real-time data analysis offers invaluable insights into the virus’s evolutionary dynamics, aiding informed decision making for public health responses. This allows authorities to enact suitable measures, such as adjusting testing strategies and bolstering contact tracing efforts, to effectively mitigate new waves of infection and prevent rapid spread within communities.

## Data Availability

Not applicable.
